# Targeting Wnt/β-catenin-mediated upregulation of oncogenic NLGN3 suppresses cancer stem cells in glioblastoma

**DOI:** 10.1038/s41419-023-05967-x

**Published:** 2023-07-13

**Authors:** Eun-Jin Yun, Donghwi Kim, Sangwoo Kim, Jer-Tsong Hsieh, Seung Tae Baek

**Affiliations:** 1 POSTECH Biotech Center, POSTECH, Pohang, Republic of Korea; 2 Department of Life Sciences, POSTECH, Pohang, Republic of Korea; 3 Department of Biomedical Systems Informatics and Brain Korea 21 PLUS Project for Medical Science, Yonsei University College of Medicine, Seoul, Republic of Korea; 4 Department of Biotechnology, Kaohsiung Medical University, Kaohsiung, Taiwan Republic of China; 5 Department of Urology, University of Texas Southwestern Medical Center, Dallas, TX USA

**Keywords:** Cancer stem cells, CNS cancer

## Abstract

Glioblastoma (GBM) is the most malignant tumor in brain and is highly resistant to therapy. Clinical evidence suggests increased number of cancer stem cells (CSCs) may contribute to the failure of conventional therapies, but the mechanisms associated with acquisition of CSC properties in GBM are not fully understood. We found that DAB2IP suppresses CSC properties by targeting the synaptic proteins neuroligin 3 (NLGN3) in GBM. Furthermore, we showed that GBM-derived NLGN3 has an oncogenic function by inducing CSC properties within GBM. Moreover, elevated NLGN3 transcription mediated by Wnt/β-catenin signaling pathway resulted in increased secretion of NLGN3 into the surrounding tumor microenvironment. Both condition media containing NLGN3 and recombinant NLGN3 transformed neighboring cells to CSCs, suggesting NLGN3 as a critical component inducing CSC properties. Furthermore, targeting NLGN3-bearing CSCs using upstream Wnt/β-catenin inhibitors synergistically enhances the efficacy of conventional treatment. Hence, we unveiled the series of regulatory mechanisms for acquisition of CSC properties in GBM progression by Wnt/β-catenin-mediated NLGN3. These results may provide a new targeting strategy to improve the therapeutic efficacy of GBM treatments.

## Introduction

Standard therapy for Glioblastoma (GBM) includes maximal surgical resection followed by concurrent radiation therapy with adjuvant temozolomide (TMZ) therapy [1, 2]. Although many tumors initially respond to the treatment, the current regimen cannot prevent inevitable tumor recurrence; thus, GBM remains the most prevalent and lethal primary malignant brain tumor [1]. Accumulating evidence suggests that cancer stem cells (CSCs) characterized by inter- and intra-tumoral heterogeneity are largely responsible for treatment failure mainly due to their enhanced tumorigenicity and chemoresistance [3, 4]. Therefore, the importance of CSCs has been emphasized in GBM as well as other solid malignancies, and understanding the underlying mechanisms involved in GBM-CSCs and therapeutic resistance could lead to new strategies for treatment of GBM [5–7].

DOC-2/DAB2 interactive protein (DAB2IP) is characterized as tumor suppressor and showed critical role in suppressing CSCs properties in some cancer types [8–10]. In this study, we observed that loss of DAB2IP in GBM exhibited CSCs properties. To study GBM-CSCs related mechanisms, we used DAB2IP knock-down and overexpression systems as models. By performing RNA-sequencing with DAB2IP modulated GBM cell lines, we newly identified neuroligin 3 (NLGN3) as a novel regulator in maintaining CSCs. NLGN3 is postsynaptic protein that interact with presynaptic neurexin 3 (NRXN3) to mediate synapse formation and trans-synaptic signaling [11, 12]. In addition to its physiological function in synapse formation, recent studies have suggested oncogenic function of NLGN3 in brain cancer [13–15]. Neuronal activity-induced cleavage of NLGN3 from neurons and oligodendrocyte precursor cells (OPC) promoted glioma proliferation through PI3K-mTOR pathway, and NLGN3 derived from neurons and OPC stimulate feed forward expression of NLGN3 in cancer cell [13, 14]. In this study, we observed overexpression of NLGN3 transformed not only modulated cells themselves, but also other neighbor cells into more cancer stem-like properties. In particular, unlike in previous report, GBM cells secreted full length NLGN3 rather than cleaved form.

We further demonstrated comprehensive inhibitory mechanisms of DAB2IP on NLGN3 regulation that underlined the CSCs properties associated with therapeutic resistance. Mechanistically, DAB2IP-mediated suppression of Wnt/β-catenin signaling pathway resulted in decrease of NLGN3 transcription. Consistent with these findings, treatment of Wnt/β-catenin inhibitor suppressed NLGN3 expression, and combination therapy using temozolomide and Wnt/β-catenin inhibitor showed significantly enhanced therapeutic efficacy of GBM.

In conclusion, this study demonstrated that NLGN3 which is elevated in malignant GBM cell lines plays a critical role in CSCs properties in which Wnt/β-catenin signaling pathway is further defined as a key underlying mechanism. These findings suggest NLGN3 as a potential prognostic marker and also provides new therapeutic strategy targeting both CSCs and its non- progeny CSCs.

## Results

### Loss of *DAB2IP* is associated with stem cell-like properties in GBM cells and poor clinical outcomes in patients

**Fig. 1:**
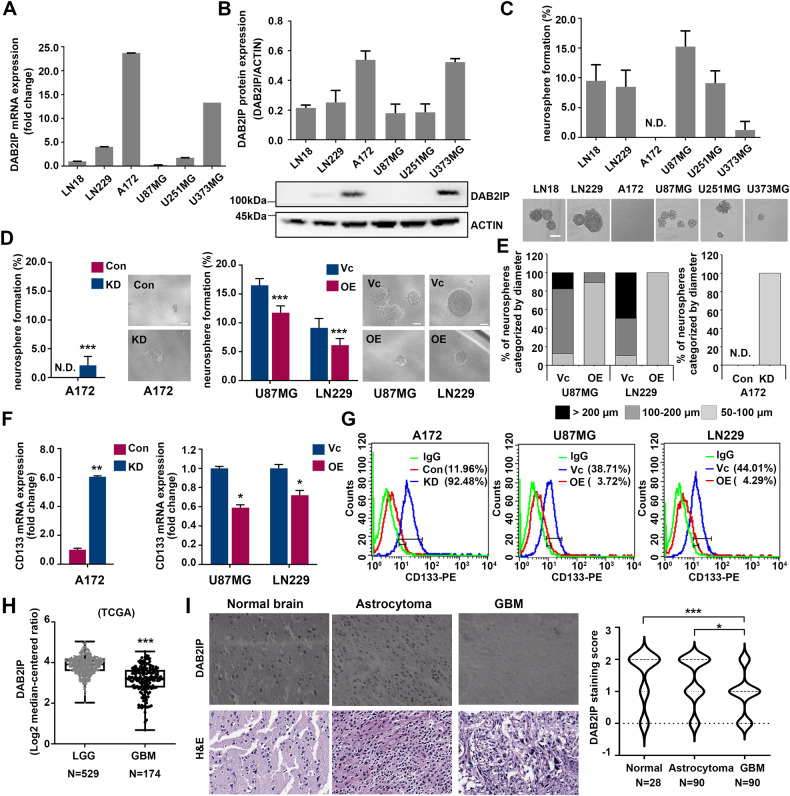
Loss of DAB2IP is associated with cancer stem cell properties in GBM. **A** The mRNA expression of DAB2IP in various glioblastoma cell lines were analyzed by qRT-PCR. After normalizing with 18 S rRNA in each sample, the relative mRNA levels were calculated by setting the expression level in LN18 to 1. Means ± SD; n = 3. **B** We analyzed the relative expression levels of DAB2IP protein in glioblastoma cell lines, normalized to ACTIN, by graphing the results from three replications of western blotting (top). The representative images of western blot against DAB2IP and β-actin were shown below (bottom). **C** Cells were cultured with sphere forming condition in ultra-low attachment plate for 14 days. The numbers of spheres larger than 50 µm were counted, and sphere forming ability was calculated based on the seeding number. N.D., not detectable. **D** Sphere forming ability were analyzed in DAB2IP modulated glioblastoma cell lines. Red and blue bars indicate DAB2IP-high and DAB2IP-low expression cell lines, respectively. Con, control cells; KD, DAB2IP knock-down cells; Vc, vector control cells; OE, DAB2IP overexpression cells; N.D., not detectable. Means ± SD; n = 16; Student’s two tailed t-test, ***p < 0.001. Scale bar=100 µm. **E** The size of neurospheres in diameter was measured, and the percentage of each sphere was calculated. A172 Con didn't form any size of spheres. N.D., not detectable. **F** The expression level of CD133 mRNA was analyzed by qRT-PCR. After normalizing with 18 S rRNA in each sample, the relative mRNA levels were calculated by setting the expression level in Con or Vc to 1. Means ± SD; n = 3; Student’s two tailed t-test, *p < 0.05, **p < 0.01. **G** Cells were stained with PE-conjugated CD133 and analyzed by flow cytometry. **H** The levels of DAB2IP mRNA expression in low grade glioma (LGG) versus glioblastoma (GBM) tissues. The results shown here are in whole based upon data generated by the TCGA Research Network: https://www.cancer.gov/tcga. Unpaired t-test, ***p < 0.001. **I** Representative TMA of DAB2IP in clinical specimens (left). The scale bar represents 100 µm. Right: DAB2IP staining score from 0–3 were analyzed in different tissues. One-way ANOVA, **p < 0.01, ***p < 0.001.

To determine the expression pattern of DAB2IP, we investigated mRNA and protein levels in several GBM cell lines, including A172 and LN229. The results showed that A172 and U373 highly expressed DAB2IP, whereas other GBM cell lines, including LN18, LN229, U87MG, and U251, lost DAB2IP expression (Fig. 1A, B). Interestingly, we found that the expression level of DAB2IP was opposite to the neurosphere-forming ability, which was representative of the self-renewal potential in vitro (Fig. 1C).

To investigate the effects of DAB2IP on GBM, we stably modulated DAB2IP expression in A172, U87MG, and LN229 cell lines (Fig. S1A). Although no morphological changes were observed in all cells following modulation of DAB2IP expression, a reduction of DAB2IP resulted in a relatively accelerated cell growth (Fig. S1B, C). Additionally, following knock-down (KD) of *DAB2IP*, A172 cells exhibited a more malignant phenotype with increased colony formation and invasion (Fig. S1D, E). In contrast, DAB2IP overexpression (OE) in both U87MG and LN229 significantly decreased colony formation abilities and invasiveness (Fig. S1D, E). Furthermore, DAB2IP-low cells (A172 KD, U87MG Vc, and LN229 Vc) could form more and bigger spheres under three-dimensional suspension conditions (Fig. 1D). Since self-renewal is a representative feature of CSCs, sphere formation assays are often used to identify CSCs in solid tumors [16, 17]. A172 was originally unable to form a single sphere, but after knocking down DAB2IP, about 2% of seeded cells formed neurospheres (Fig. 1D). In addition to the number of spheres, DAB2IP also affects the size of formed spheres (Fig. 1E). About 90% of the formed spheres had a diameter of over 100 µm in U87MG and LN229, overexpression of DAB2IP in these cells not only decreased the number of spheres, but most of the formed spheres also showed small size of 100 µm or less (Fig. 1E). Next, we screened various stem cell markers and found the GBM-CSCs surface marker, CD133, to be increased in all DAB2IP-low cells, suggesting that DAB2IP might be involved in CSCs regulation in GBM (Fig. 1F, G). Unlike CD133, other stem cell markers including CD24, CD44, and CD117 expression varied depending on the cell line (Fig. S2).

Consistent with the results in the GBM cell lines, clinical outcomes of GBM patients also showed inverse associations between GBM progression and DAB2IP expression (Fig. 1H, I). Analysis of The Cancer Genome Atlas (TCGA) database, low-grade glioma including grade I pilocytic through grade III anaplastic patients expressed significantly higher levels of DAB2IP mRNA than high-grade GBM patients (Fig. 1H). Furthermore, there were lower levels of DAB2IP expression in GBM tissue than in normal brain tissue, which complemented our in vitro results (Fig. 1I).

From these findings, we hypothesized that DAB2IP is a potential regulator suppressing GBM cells from acquiring CSCs properties.

### Synaptic proteins NLGN3 and NRXN3 are novel DAB2IP target genes in GBM cells

**Fig. 2:**
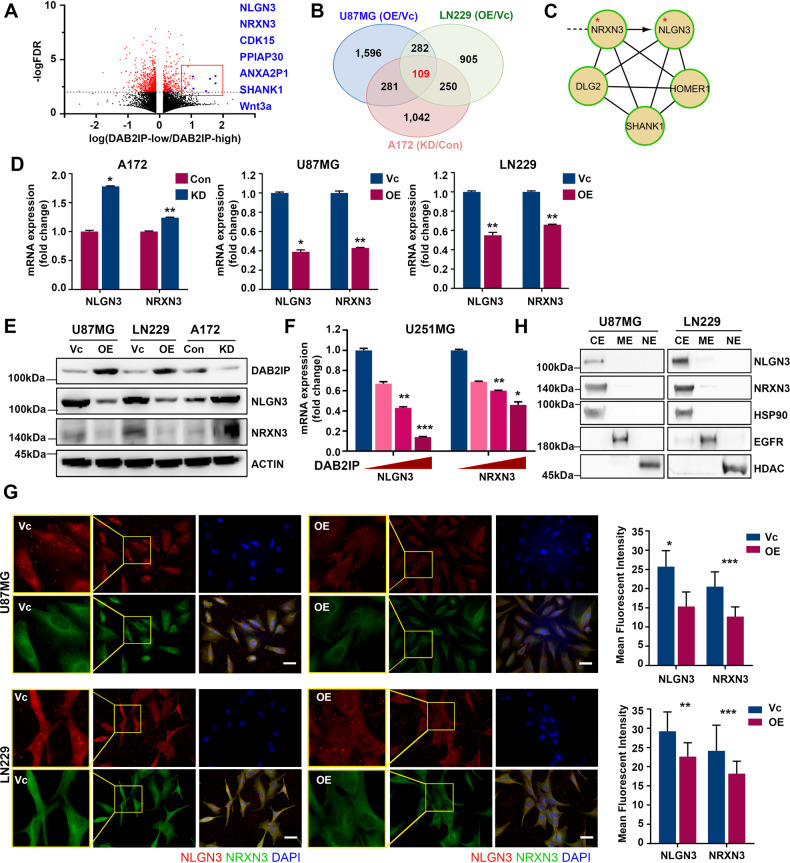
DAB2IP suppresses synaptic protein NLGN3 and NRXN3 expression in GBM. **A** Volcano plot of false discovery rate (-log10FDR) against fold change between DAB2IP-high and DAB2IP-low U87MG cells from RNA sequencing data (HiSeq 2000 platform, Illumina) was summarized. Some DEG including NLGN3 and NRXN3 are highlighted. **B** Venn diagram illustrates overlapping alternated-genes among U87MG, LN229, and A172 cells after DAB2IP modulation. **C** DAB2IP target candidate genes (109 genes) overlapped in U87MG, LN229, and A172 were analyzed using Reactome and highly interconnected networks were constructed. **D** The expression levels of NLGN3 and NRXN3 mRNA in DAB2IP modulated cells were analyzed by real-time PCR. Red and blue bars indicate DAB2IP-high and DAB2IP-low expression cell lines, respectively. Means ± SD; Student’s two tailed t-test, *p < 0.05, **p < 0.01. **E** NLGN3 and NRXN3 protein expressions were compared between DAB2IP-high and –low cells by western blot analysis. **F** After U251 cells were transfected with increment amount of DAB2IP for 48 h, NLGN3 and NRXN3 mRNA expressions were analyzed by real-time PCR. Means ± SD; Student’s two tailed t-test, *p < 0.05, **p < 0.01, ***p < 0.001. **G** Intracellular localization of NLGN3 and NRXN3 proteins were determined by immunocytochemistry, and expression patterns were compared in DAB2IP-high and -low cells. Scale bar=10 µm. Mean fluorescent intensity of NLGN3 or NRXN3 was analyzed using Image J. Means ± SD; Student’s two tailed t-test, *p < 0.05, **p < 0.01, ***p < 0.001. **H** U87MG and LN229 cells were fractionated into cytosol extract (CE), membrane extract (ME), and nuclear extract (NE), then the expressions of NLGN3 and NRXN3 protein were compared in each fraction.

DAB2IP plays a critical role in suppressing stemness through various mechanisms in other cancer types [8–10]. To investigate the molecular mechanism underlying the effects of DAB2IP regulating GBM-CSCs properties, we performed RNA-sequencing (RNA-seq) using DAB2IP modulated GBM cell lines (A172 Con/KD, U87MG Vc/OE, LN229 Vc/OE) and obtained potential DAB2IP target genes, which were commonly altered by DAB2IP in all cell lines (Fig. 2A, B, Fig. S3). We then analyzed 109 common candidate genes in three cell lines based on the highly interconnected networks using Reactome [18]. These genes were involved in various biological processes, including signal transduction, cell proliferation, and extracellular matrix organization. Interestingly, gene ontology (GO) enrichments analyses identified a group of genes involved in synapse assembly and organization, including NLGN3 and NRXN3, thus we focused the role of DAB2IP on regulating synaptic genes (Fig. 2C).

Consistent with the results from RNA-seq, knock-down of *DAB2IP* increased both NLGN3 and NRXN3 expression in A172 cells, whereas DAB2IP overexpression decreased their expression in U87MG and LN229 (Fig. 2D, E). Transient overexpression of DAB2IP with increased plasmid concentration in U251 also resulted in dose-dependent suppression of NLGN3 and NRXN3 expression, suggesting that they were DAB2IP downstream target genes (Fig. 2F). We further validated these results using immunofluorescent staining to show NLGN3 and NRXN3 expression patterns in GBM. As expected, DAB2IP overexpressing cells decreased both NLGN3 and NRXN3 expression (Fig. 2G). However, unlike neurons, subcellular localization of NLGN3 and NRXN3 were not membrane-specific, but instead spread throughout the cytosol in GBM cells (Fig. 2G). To confirm this unexpected finding, these cells were fractionated into cytosolic, membrane, and nuclear compartments, and western blot analyses were conducted. As shown in Fig. 2H, NLGN3 and NRXN3 were primarily expressed in cytosolic compartments rather than in the membrane, suggesting that their roles in cancer cells might be different from those in neurons.

### NLGN3 plays a role in maintaining stem cell-like properties of GBM cells

**Fig. 3:**
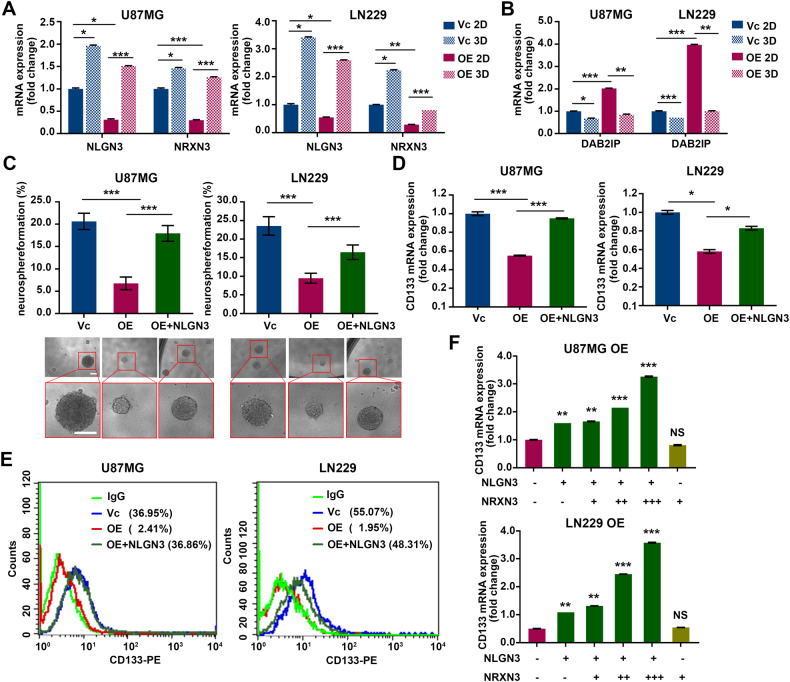
NLGN3 plays a role in maintaining cancer stem cell properties of GBM cells. NLGN3, NRXN3 mRNA (**A**) and DAB2IP (**B**) expressions were compared between monolayer (2D) and sphere (3D) culture condition. Red and blue bars indicate DAB2IP-high and DAB2IP-low expression cell lines, respectively. Means ± SD; One-way ANOVA, *p < 0.05, **p < 0.01, ***p < 0.001. **C** After restoration of NLGN3 expressions in U87MG OE (DAB2IP-high, NLGN3-low) and LN229 OE (DAB2IP-high, NLGN3-low) cells, sphere forming abilities including both numbers and size were compared. Red and blue bars indicate DAB2IP-high and DAB2IP-low expression cell lines, green bar indicates NLGN3 restoration in DAB2IP-high cells, respectively. Means ± SD; One-way ANOVA, ***p < 0.001. Scale bar=100 µm. **D** After restoration of NLGN3 expressions in U87MG OE and LN229 OE cells, CD133 mRNA expression was compared. Means ± SD; One-way ANOVA, *p < 0.05, ***p < 0.001. **E** After restoration of NLGN3 expressions in U87MG OE and LN229 OE cells, cells were stained with PE-conjugated CD133 and analyzed by flow cytometry. **F** U87MG OE and LN229 OE cells were co-transfected with NLGN3 and increment amount of NRXN3, and CD133 mRNA expression was compared. Means ± SD; One-way ANOVA, **p < 0.01, ***p < 0.001.

NLGN3 and NRXN3 belong to the synaptic protein family, and the trans-synaptic interaction between presynaptic NRXNs and postsynaptic NLGNs are necessary to form a functional synapse in brain [19, 20]. In addition to synaptic functions, NLGN3 has oncogenic functions, which promote the proliferation of high-grade glioma [13, 14]. In the GBM cell line, we found that the expressions of NLGN3 and NRXN3 were significantly enriched in neurospheres under sphere culture conditions (3-dimensional, 3D condition) compared to typical adherent culture conditions (2-dimensional, 2D condition) in normal media containing 10% FBS (Fig. 3A, B). On the other hand, DAB2IP expression was decreased in neurospheres under 3D conditions because DAB2IP overexpressing cells cannot for spheres (Fig. 3B). While NLGN3 and NRNX3 expression decreased in DAB2IP overexpressing cells under the 2D culture condition, their expression was maintained in the already formed neurospheres (Fig. 3A). Based on these observations, we hypothesized that NLGN3 and NRXN3 increased their expression in a more stem-like sphere condition, and investigated how synaptic proteins were involved in GBC-CSCs regulation. We conducted rescue experiments to investigate the role of NLGN3 and NRXN3 on GBM-CSCs properties which were altered by DAB2IP. Consistent with previous tests, the overexpression of DAB2IP reduced self-renewal, including reductions in both sphere numbers and sphere size; however, rescue of NLGN3 in DAB2IP overexpressing cells significantly recovered their sphere-forming ability in both U87MG and LN229 cell lines (Fig. 3C). Similarly, NLGN3 overexpression also recovered the expression of the stem cell marker, CD133, in both mRNA and surface protein levels, suggesting the involvement of NLGN3 in regulating GBM-CSCs properties (Fig. 3D, E). Unlike with NLGN3, the overexpression of NRXN3 did not affect either sphere formation or CD133 expression in DAB2IP overexpressing cells (Fig. S4A, B). However, the co-occurrence of NLGN3 and NRXN3 showed more effective recovery of CD133 expression than in the overexpression of NLGN3 alone (Fig. 3F and Fig. S4C). These results suggest that NLGN3 plays a critical role in regulating GBM-CSCs and that NRXN3 may act as a co-factor of NLGN3 function in GBM.

### NLGN3 secretion promotes CSCs properties of neighboring cells

**Fig. 4:**
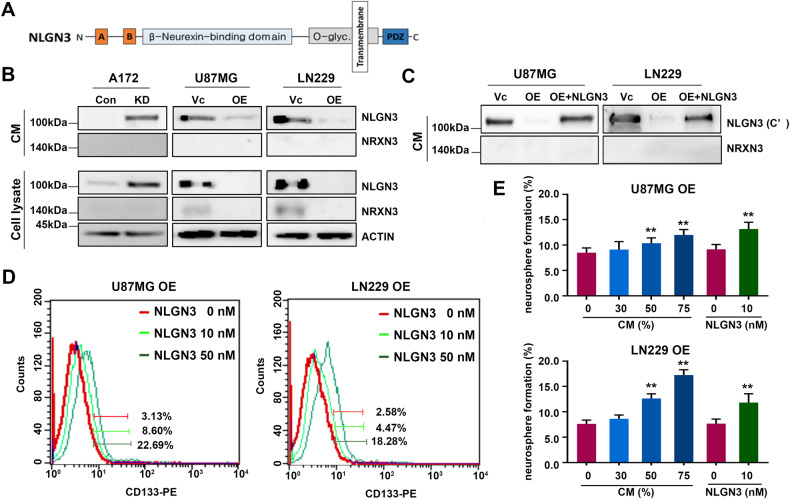
Secreted NLGN3 plays a role in maintaining cancer stem cell properties of GBM cells. **A** Scheme of NLGN3 functional domains. **B** NLGN3 and NRXN3 protein expressions were determined in cell lysates and CM derived from DAB2IP-high and –low cells. Primary NLGN3 antibody (NovusBio) detecting N-terminal epitope of NLGN3 was used. **C** NLGN3 and NRXN3 protein expressions were determined in CM derived from DAB2IP-high and –low cells. Primary NLGN3 antibody (Abcam) detecting C-terminal epitope of NLGN3 was used. **D** Cells treated with recombinant NLGN3 for 48 h were stained with PE-conjugated CD133 and analyzed by flow cytometry. PE-IgG was used as the negative control for gating and the labels indicated the percentage of each cell population. **E** U87MG OE and LN229 OE cells were cultured in ultra-low attachment plate under sphere forming culture condition for 14 days, and CM media derived from U87MG Vc and LN229 Vc cells or recombinant NLGN3 protein were added into sphere every 3 days. Means ± SD; One-way ANOVA, **p < 0.01.

NLGN3 consists of a large extracellular N-terminal ectodomain involved in trans-synaptic interactions with NRXN3 and a short C-terminal cytoplasmic domain exposed to a variety of intracellular regulatory signals (Fig. 4A) [11, 21]. In neurons, NLGN3 is secreted by enzymatic cleavage of the N-terminal ectodomain, and this secreted form acts as a mitogen to neighboring glioma cells [13]. To determine whether increased NLGN3 expression in GBM cell lines resulted in increased secretion, we collected conditioned medium (CM) after 72 h-cultivation without serum. A172 KD, U87MG Vc, and LN229 Vc cells which expressed low levels of DAB2IP, showed increased NLGN3 expression in both the cell lysate and CM (Fig. 4B). Interestingly, unlike CM from neurons, we detected NLGN3 using antibodies against cytoplasmic C-terminal epitopes, suggesting that CM from GBM contained full-length NLGN3 (Fig. 4B, C). Based on these results and previous findings that NLGN3 was primarily expressed in the cytoplasmic compartment of GBM (Fig. 2G, H), we assumed that the secretion mechanisms of NLGN3 were different in GBM and neurons. To further investigate the effects of exogenous NLGN3 on GBM we used CM derived from cells with high levels of NLGN3 or recombinant NLGN3. When exposed to exogenous NLGN3, DAB2IP overexpressing cells, which expressed low levels of NLGN3, showed increased CD133 expression as well as neurosphere formation in a dose-dependent manner (Fig. 4D, E and Fig. S5). These findings indicated that GBM cells secrete cytosolic full-length NLGN3 that induces GBM-CSCs properties in neighboring cells.

### DAB2IP regulates NLGN3 transcription via Wnt/β-catenin signaling pathway

**Fig. 5:**
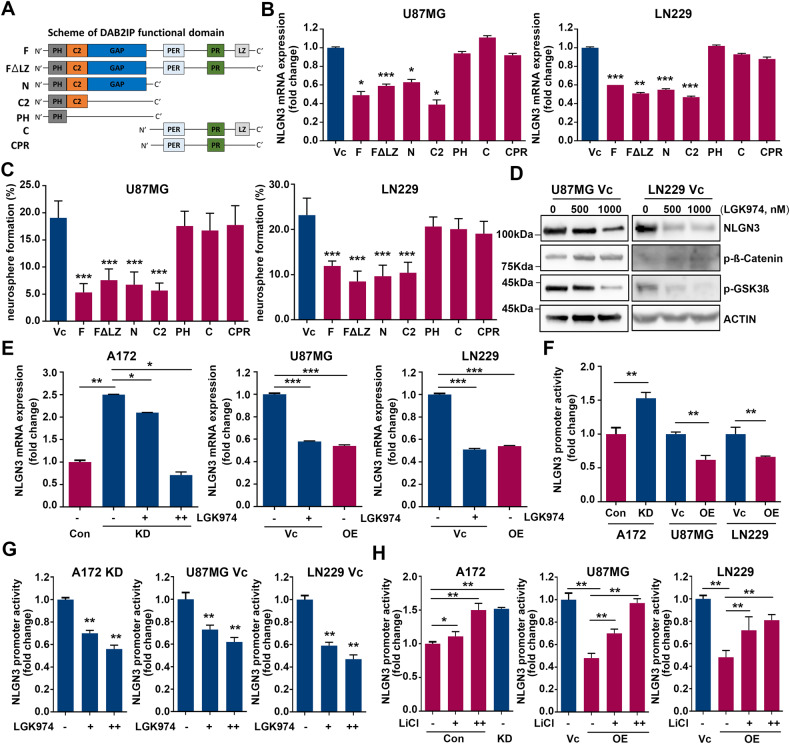
DAB2IP regulates NLGN3 transcription through Wnt/β-catenin signaling pathway. **A** Scheme of DAB2IP functional domains. **B** Cells were transfected with various functional domains of DAB2IP and NLGN3 mRNA expressions were determined. Means ± SD; One-way ANOVA, *p < 0.05, **p < 0.01, ***p < 0.001. **C** Cells were transfected with various functional domains of DAB2IP, and cultured in ultra-low attachment plate under sphere forming condition. Sphere forming ability was calculated based on seeding cell numbers. Means ± SD; One-way ANOVA, ***p < 0.001. **D** Cells were treated with Wnt/β-catenin inhibitor (LGK974), and NLGN3 protein expressions were compared. **E** DAB2IP-low cells (A172 KD, U87MG Vc, and LN229 Vc) were treated with LGK974 and NLGN3 mRNA expression was compared. Means ± SD; One-way ANOVA, *p < 0.05, **p < 0.01, ***p < 0.001. **F** DAB2IP modulated cells were transfected with NLGN3 promoter containing luciferase and reporter activity was compared by dual luciferase assay. Means ± SD; One-way ANOVA, **p < 0.01. **G** DAB2IP-low cells transfected with NLGN3 promoter were treated with LGK974 and reporter activity was determined by dual luciferase assay. Means ± SD; One-way ANOVA, **p < 0.01. **H** DAB2IP-high cells transfected with NLGN3 promoter were treated with Wnt/β-catenin agonist, LiCl and reporter activity was determined by dual luciferase assay. Means ± SD; One-way ANOVA, *p < 0.05, **p < 0.01, ***p < 0.001.

To investigate the key mechanisms regulating NLGN3, we utilized plasmids containing different functional domains of DAB2IP (Fig. 5A). U87MG and LN229 cells were transfected with full-length (F), FΔLZ, N-terminal (N), C-terminal (C), C2 domain (C2), PH domain (PH), and PER-PR domain (CPR) constructs of DAB2IP for 48 h and their NLGN3 mRNA expression was examined. As shown in Fig. 5B, most DAB2IP plasmids significantly decreased NLGN3 mRNA expression, while PH, C, and CPR constructs lacking the C2 domain lost their inhibitory function, suggesting that the C2 domain was required to inhibit NLGN3 expression (Fig. 5B). Consistently, DAB2IP constructs containing the C2 domain inhibited neurosphere formation in both U87MG and LN229 cells (Fig. 5C). Since the C2 domain in DAB2IP is known to act as a scaffold protein recruiting PP2A to the APC-GSK3β-Axin complex in the Wnt/β-catenin signaling pathway, we further confirmed the involvement of Wnt/β-catenin signaling in NLGN3 regulation by DAB2IP [22]. The treatment of Wnt/β-catenin inhibitor, LGK974, decreased NLGN3 expression even in DAB2IP-low cell lines (Fig. 5D, E). Mechanistically, DAB2IP was able to inhibit *NLGN3* gene transcription that promoter activity increased in A172 KD cells, whereas it decreased in both U87MG OE and LN229 OE cells (Fig. 5F). Moreover, the relative transcriptional activities of *NLGN3* in DAB2IP-low cells were significantly decreased after treatment with the Wnt/β-catenin inhibitor while Wnt/β-catenin agonist, LiCl treatment increased *NLGN3* promoter activity in DAB2IP-high cells in a dose-dependent manner (Fig. 5G, H). Upon examining RNA-seq date after DAB2IP modulation, an increase in the mRNA expression of Wnt3a was observed in DAB2IP-low cells (Fig. 2A). Therefore, we next investigated the impact of Wnt ligands on the expression of NLGN3, and observed that treatment with Wnt3a for 12 hours significantly increased the NLGN3 mRNA expression in DAB2IP-high cells of both U87MG and LN229. In contrast, this effect was almost negligible in DAB2IP-low cells under the same experimental condition suggesting there might be an adequate level of Wnt ligands already present in the culture environment of DAB2IP-low cells (Fig. S6A). Similar to the results from NLGN3 regulation, the C2 domain in DAB2IP was required to inhibit *NRXN3* mRNA expression, and LGK974 treatment inhibited the transcriptional activity of *NRXN3* (Fig. S6B, C). This suggested that Wnt/β-catenin is the key signaling pathway that regulates *NLGN3* and *NRXN3* transcription.

### Wnt/β-catenin inhibition enhances in vivo therapeutic efficacy on GBM tumors

**Fig. 6:**
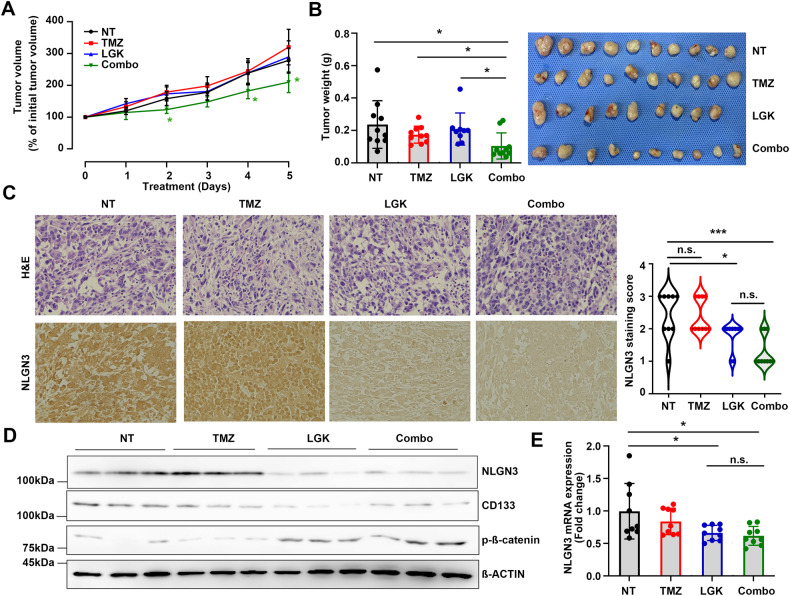
Blocking Wnt/β-catenin signaling enhances therapeutic efficacy in GBM. **A** GL261 cells were subcutaneously injected into C57/BL6 mice. When tumors became palpable, the mice were treated with TMZ (25 mg/kg/i.p.), LGK974 (3 mg/kg/oral gavage), or combination daily and tumor sizes were measured. **B** After treatment for 5 consecutive day, all tumors were excised and photographed. Means ± SD; n = 10; Student’s two tailed t-test, *p < 0.05. **C** The expression levels of NLGN3 in tumors were analyzed by IHC, and representative results were displayed. The scale bar represents 100 µm. NLGN3 staining score from 0–3 were analyzed in tumor tissues. One-way ANOVA, *p < 0.05, ***p < 0.001. The expression levels of NLGN3 mRNA (**D**) and protein (**E**) were analyzed. Means ± SD; One-way ANOVA, *p < 0.05.

TMZ is a first-line chemotherapeutics used as an adjuvant in radiotherapy for GBM patients, however, over 50% of GBM patients treated with TMZ do not respond to the therapy [23]. We hypothesized that resistance to TMZ might be due to residual CSCs after treatment, and further demonstrated the effect of targeting enriched CSCs using Wnt/β-catenin inhibitor combining with TMZ. We employed a mouse syngeneic tumor model carrying GL261 tumors which showed high TMZ-resistance with the IC_50_ ranged 1,458 ± 357.5 μM in vitro (Fig. S7A). Consistent with the results based on our hypothesis and findings, GL261 cells treated with combination regimen showed synergistic effect (CI = 0.58) in vitro as well as in vivo treatment (Figs. S7B and 6A). Treatment with either TMZ (25 mg/kg) or LGK974 (3 mg/kg) alone had no effect, but combination therapy targeting both CSC population and fast-growing cells showed a significant inhibition of tumor growth compared with mice treated with single agent suggesting the combination of a Wnt/β-catenin inhibitor with TMZ as a novel therapeutic strategy to overcome TMZ-resistance (Fig. 6A, B). Also, decreased NLGN3 expression was noticed in LGK974 or combination treatment groups, whereas single treatment of TMZ failed to suppress NLGN3 and CD133 expression which may explain the therapeutic failure of TMZ (Fig. 6C, D). We further confirmed decreased NLGN3 mRNA levels in tumors harvested from LGK974 or combination treatment (Fig. 6E) indicating blockade of Wnt/β-catenin signaling suppressed NLGN3 transcription in GL261 tumors, consistent with previous in vitro results.

**Fig. 7:**
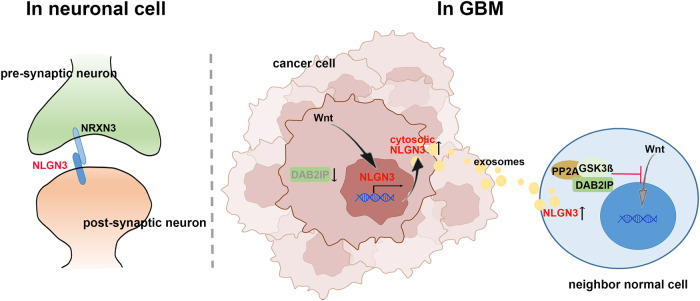
The model of mechanism regulating GBM by NLGN3. In GBM, cytoplasmic NLGN3 is secreted extracellularly and induces oncological function in neighboring cells.

In conclusion, DAB2IP suppressed GBM-CSCs properties by blocking Wnt/β-catenin signaling pathway (Fig. 7). Therefore, utilizing a Wnt/β-catenin inhibitor targeting GBM-CSCs synergize the effect of conventional TMZ therapy on eradicating proliferative CSCs progeny in GBM.

## Discussion

Since CSCs are enriched after treatment with conventional therapies, strategies to reduce the expression of stemness-related genes may be a way to overcome therapeutic resistance [3, 4]. In this regard, here we focused on identifying key molecules involved in GBM-CSCs regulation to better understand the unique environment of GBM and develop novel therapeutic strategies for therapy-resistant GBM.

In this work, we have discovered that DAB2IP was inversely correlated with GBM progression and was associated with CSCs properties in GBM. DAB2IP suppresses the development of CSCs by introducing different regulatory mechanisms in various types of cancer [8–10, 24, 25]. Additionally, we report *NLGN3* and *NRXN3* as novel downstream targets of DAB2IP involving GBM-CSCs development. NLGN3 and NRXN3 are synaptic cell-adhesion molecules that form a functional synapse in the brain [19, 26]. In addition to their synaptic function, Venkatesh et al. [13, 14] recently showed the oncogenic function of NLGN3 is stimulated by neuronal activity in high-grade glioma. However, the role of GBM-derived NLGN3 has not been studied yet. In this study, we found that NLGN3 expression was elevated in aggressive GBM tissues and cell lines, suggesting that GBM-derived NLGN3 might also be involved in oncogenic processes. The overexpression of NLGN3 in GBM not only promoted cell growth, but also increased its CSCs properties, including neuro-sphere formation and CD133 expression.

NLGN3 is primarily expressed on the postsynaptic terminal of excitatory synapses, but the N-terminal ectodomain of NLGN3 in neurons and OPCs can also be secreted by enzymatic cleavage by the ADAM metallopeptidase domain 10 (ADAM10) [14], and then the cleaved form of NLGN3 promotes glioma cell proliferation through the PI3K-mTOR signaling pathway [13]. Contrary to these reports, we observed that NLGN3 expressed in GBM cells was secreted into the media in its full-length form rather than the cleaved form. Unlike secretions through the proteolytic mechanism in OPCs and neurons, GBM-derived NLGN3 was secreted as full-length form.

In addition, we discovered some interesting features of NRXN3. Unlike NLGN3, overexpression of NRXN3 alone did not affect CSC properties of GBM. However, the co-expression of NRXN3 synergized the effects of NLGN3 to promote CSC properties, suggesting that NRXN3 may act as a co-factor of NLGN3 function in GBM. To gain further insight into the role of NRXN3 in GBM-CSCs regulation, it is critical to dissect the clear mechanism of interactions between NLGN3 and NRXN3. Furthermore, since high doses of NLGN3 are toxic to GBM cells under three-dimensional culture conditions and the efficacy of recombinant NLGN3 affect each cell differently (A172, LN229, and U87MG cells), it suggests that other effective factors may exist depending on the tumor environment; this requires further investigation.

In terms of the upstream regulatory mechanism of NLGN3 and NRXN3, as the expression of *NLGN3* and *NRXN3* mRNA was inhibited by DAB2IP, we focused on the pathway encompassing DAB2IP and the expression of these synaptic proteins. Many signaling pathways, such as Wnt/β-catenin, PI3K/Akt/mTOR, Notch, Hedgehog, and Receptor Tyrosine Kinase (RTK), play important roles in CSCs [27–32]. In this study, we showed that the inhibition of the Wnt/β-catenin pathway by LGK974 inhibited both the promoter activity and mRNA expression of *NLGN3* and *NRXN3*, suggesting the involvement of Wnt/β-catenin in the transcription of *NLGN3* and *NRXN3*. As DAB2IP has been shown to suppress the Wnt/β-catenin signaling pathway by acting as a scaffold protein [22], we assumed that DAB2IP acts as a major upstream regulator of NLGN3 and NRXN3 through the Wnt/β-catenin pathway. This DAB2IP-Wnt/β-catenin-NLGN3/NRXN3 network affecting GBM-CSC properties may help to develop potential therapeutic strategy by targeting Wnt/β-catenin to overcome therapy resistant GBM. In a previous study, we showed that the Wnt/β-catenin pathway played an important role in autophagy-related drug-resistance in GBM and that blocking it significantly decreased drug resistance [33]. Here, in vivo anti-tumorigenic effect of blocking Wnt/β-catenin was tested using GL261 murine GBM cell line. Although GL261 may not perfectly mimic the characteristics of human GBM, the experimental strategy employed in this study could still offer valuable insights into the potential therapeutic benefits of targeting the Wnt/β-catenin pathway in GBM, and could serve as a basis for further investigations in clinically relevant model. Together, we showed that the Wnt/β-catenin pathway plays a key role in the progression of GBM, including CSCs properties, and once again supports the possibility that targeting Wnt/β-catenin pathway will be a breakthrough in GBM therapy.

## Materials and Methods

### Cell culture (2-dimensional culture condition)

Human glioblastoma cell lines, LN18 and LN229, were purchased from ATCC and cultured in Dulbecco’s Modified Eagle’s Medium (DMEM, Lonza, Basel, Switzerland) containing 5% fetal bovine serum (FBS) and antibiotics (penicillin/streptomycin 100 IU/mL, Gibco, MA, USA) at 37 °C in 5% CO_2_. Human glioblastoma cell lines A172, U87MG, U251, and U373 were provided by Dr. Kyu Lim (College of Medicine, Chungnam National University, Korea) [34]. These cell lines were maintained in DMEM containing 10% FBS and antibiotics at 37 °C in a 5% CO_2_ atmosphere. All cell lines were mycoplasma free and routinely tested by PCR amplification.

### Plasmid construction and reagents

The DAB2IP expression plasmid was prepared as described previously [22], and stable clones were selected by G418 at 1000 µg/mL for 3–4 wk. Knock-down of *DAB2IP* gene was performed using pGIPZ-DAB2IP-lentiviral-shRNAmir and pGIPZ-non-silencing-lentiviral-shRNAmir purchased from Open Biosystems, according to the manufacturer’s protocol. Following infection, cells were selected by puromycin at 0.2 µg/mL for 3–4 wk. The NLGN3 expression plasmid (CAG-HA-NLGN3 WT) was acquired from Peter Scheiffele (Addgene plasmid #59318; http://n2t.net.addgene:59318; RRID:Addgene_59318) [35]. Primary antibodies used were as follows: anti-NLGN3 (NBP1–90080, N-terminal epitope) was purchased from NOVUS biologicals; anti-DAB2IP (ab87811), anti-NLGN3 (ab186307, C-terminal epitope), and anti-NRXN3 (ab230635) were from Abcam; and anti-EGFR (Cat#4267), anti-HDAC2 (Cat#57156), anti-HSP90 (Cat#4874), anti-phospho-β-Catenin (Cat#9565), anti-phospho-GSK-3β (Cat#5558), and anti-actin (Cat#4970) were purchased from Cell Signaling Technology. PE-conjugated mouse anti-human CD133 (566593) was purchased from BD Pharmingen.

### Immunohistochemistry (IHC) and tissue microarray (TMA)

The brain primary tumor tissue microarray (GL208) was purchased from Biomax (MD, USA). Formalin-fixed, paraffin-embedded sections were deparaffinized, rehydrated, and subjected to heat-induced antigen retrieval (10 mM citrate buffer, pH 6.0). Sections were blocked with CAS-Block reagent (Thermo Fisher Scientific, MA, USA) and incubated with the appropriate primary antibody. After blocking endogenous peroxidase activity, immunohistochemistry of DAB2IP and NLGN3 was performed using a VECTASTAIN Elite ABC HRP Kit (Vector Labs, CA, USA) according to the manufacturer’s instruction and counterstained with hematoxylin. Each sample stained with DAB2IP or NLGN3 was scored as negative (0), weak (1), intermediate (2) or strong (3) according to staining intensity.

### RNA isolation and quantitative reverse-transcription PCR (qRT-PCR)

RNAs were extracted using the ReliaPrep RNA Miniprep System (Promega, WI, USA), and 1 µg RNA was reverse transcribed with the LunaScript (NEB, MA, USA). The quantitative PCR (qPCR) analysis was performed using Luna Universal SYBR (NEB) on an MIC qPCR Cycler (BMS, Queensland, Australia). The primers were synthesized by Macrogen with sequences retrieved using NCBI primer blast. The relative level of target mRNA was evaluated using the Ct method and the fold change was determined by calculating 2^-∆∆Ct^ normalized with 18 S rRNA.

### Western blotting

Whole protein extracts were prepared with ice-cold RIPA lysis buffer supplemented with protease and phosphatase inhibitors (ATTA), and the protein from each subcellular fraction was prepared using the Subcellular Protein Fractionation kit (ThermoFishcer Scientific, Cat#78840). For western blot analysis, proteins were subjected to electrophoresis on 4–15% Mini-PROTEAN precast gels (Bio-Rad, CA, USA). Separated proteins were transferred onto nitrocellulose membranes, which were then incubated with 5% nonfat dry milk (w/v) for 1 h and washed in PBS containing 0.1% Tween 20. Membranes were then incubated with the primary antibody, and antibody binding was detected using the appropriate secondary antibody coupled with horseradish peroxidase.

### Neurosphere forming assay (3-dimensional culture condition)

The sphere forming assay was performed by seeding single cell suspensions containing 100 cells per well in ultra-low attachment flat bottom 96-well plates (Corning, NY, USA) for 2 wk. All cells were maintained in a defined serum-free medium, DMEM/F12 (Lonza), supplemented with recombinant human epidermal growth factor (EGF, 20 ng/mL, Gibco), basic fibroblast growth factor (bFGF, 20 ng/mL, Gibco), and N-2 Supplement (Gibco), which was changed every 3 d. Subsequently, the spheres larger than 50 μm were identified, and 24 wells were evaluated for each cell culture.

### Flow cytometry

The expression of the GBM CSCs surface marker, CD133, was detected by flow cytometry (FACS Calibur, BD Biosciences, NJ, USA). Cells were stained with PE-conjugated CD133 antibody or corresponding isotype controls and analyzed using flow cytometry.

### Clonogenic survival assay

Cells were plated in 6-well plates at a clonal density of 1,000 cells per well and cultured for 10 d. Colonies were fixed and stained with 4% formaldehyde in PBS containing 0.02% crystal violet for 30 min, and washed with tap water. Absorbance at 590 nm was measured after solving the crystal violet with 10% acetic acid.

### In vitro invasion assay

We coated 6.5 μm polycarbonate filters of a transwell (24-well insert; pore size 8 μm, Corning) were coated with 50 μL Matrigel. Cells were resuspended in serum-free media plated in the upper chamber (5 × 10^4^ cells/200 μL/chamber), and the lower chambers of the transwell were filled with 500 μL of serum-containing media. After cultivation for 48 h, non-invading cells on the upper surface of the membrane were removed by a cotton swab, and cells on the lower surface were stained with 1% crystal violet. Five randomly chosen areas were photographed under a microscope, and the number of stained cells were counted.

### RNA sequencing

The samples were prepared for sequencing using the ReliaPrep RNA Miniprep System (Promega) according to the manufacturer’s instructions and sequenced on a HiSeq 2000 platform (Illumina, San Diego, CA, USA). The 101-bp sequenced paired-end reads were mapped to the hg19 reference human genome using the STAR 2-pass methods [36]. HTSeq was used to count the reads aligned to each gene based on the Ensemble gene set [37]. Samples that failed in the library preparation, sequence process, or with fewer than 10 million reads sequenced were excluded. The normalized read counts were applied to principal component or clustering analysis, which was conducted using R and Cluster 3.0 and visualized via Java Treeview [37–39].

### Immunocytochemistry

Cells were washed with PBS, fixed in 4% paraformaldehyde, permeabilized with 1% Triton X-100, and blocked in 1% bovine serum albumin (BSA). Cells were then incubated with primary antibody at 4 °C overnight, followed by secondary antibodies. Nuclei were counterstained with DAPI for 1 min and then analyzed using a fluorescence microscope (Nikon, Tokyo, Japan).

### Luciferase assay

Cells were seeded at cell density of 0.5 × 10^5^ cells onto 12-well plates and transfected with NLGN3 promoter-luciferase reporter plasmid at 70% confluence. After 48 h of incubation, cells were harvested and lysed with Passive Lysis buffer (Promega), and luciferase activity was measured using the Dual-Glo Luciferase Assay System (Promega) on the Multi-Mode Microplate Reader (BioTek, VT, USA). Relative luciferase activity was normalized with Renilla luciferase activity. Each experiment was performed in triplicates.

### Cell survival assay

Cells (5 × 10^6^) were plated in 96-well plates and different concentrations of TMZ or LGK974 were treated for 48 h. In vitro cytotoxicity was measured using 3-(4,5-dimethyl-2-thiazolyl)-2,5-diphenyl-2H-tetrazolium bromide (MTT) assays according to the manufacturer’s instructions (Roche). Drug synergistic effects were determined based on combination index (CI) [40]. The CI was calculated using the formula: ${{{\mathrm{CI}}}} = \frac{{{{{\mathrm{CA}}}},{{{\mathrm{x}}}}}}{{{{{\mathrm{ICx}}}},{{{\mathrm{A}}}}}} + \frac{{{{{\mathrm{CB}}}},{{{\mathrm{x}}}}}}{{{{{\mathrm{ICx}}}},{{{\mathrm{B}}}}}}$, where C_A,X_ and C_B,X_ are the concentration of drug A and B used in combination to achieve X% drug effect. IC_X,A_ and IC_X,B_ are the concentrations for single agents to achieve the same effect. CI < 1, synergistic; CI = 1, additive; CI > 1, antagonistic effect.

### In vivo Xenografts

All experimental procedures on animals followed the guidelines and regulations approved by the POSTECH Institutional Animal Care and Use Committee (IACUC). Female C57/BL6 mice (7-wk-old) were purchased from the Hyochang Science (Republic of Korea) and acclimatized for 1 wk under specific pathogen-free conditions in standard cages. To generate murine syngeneic tumor, GL261 (5 × 10^6^) cells were subcutaneously injected into 8-week-old female C57/BL6 mice. Treatment started when tumors developed as measurable size and TMZ (25 mg/kg/i.p.), LGK974 (3 mg/kg/oral gavage), and a combination of them were treated daily for 5 consecutive days. Tumor size was measured every day and volume was calculated by using the ellipsoid formula (π/6 × length × width × depth).

### Statistical analysis

The data were expressed as mean ± SD. Statistical analysis was performed using Prism 9.2.0 software (GraphPad, CA, USA). A student’s two-tailed t-test or One-way ANOVA were used to determine statistical differences between groups; *p* < 0.05, *p* < 0.01 and *p* < 0.001 denoted statistically significant differences and were identified with asterisks.

## Supplementary information

AJ-checklist

Supplemental Figure 1.

Supplemental Figure 2.

Supplemental Figure 3.

Supplemental Figure 4.

Supplemental Figure 5.

Supplemental Figure 6.

Supplemental Figure 7.

Supplemental Figure Legends

Original Western blot

## Data Availability

The data generated in this study are available upon request from the corresponding author.
