# Supplementary figures and images for: Targeting Wnt/β-catenin-mediated upregulation of oncogenic NLGN3 suppresses cancer stem cells in glioblastoma
Source: Cell Death Dis. 2023 Jul 13;14(7):423. doi: 10.1038/s41419-023-05967-x (PMC10344874; doi:10.1038/s41419-023-05967-x)

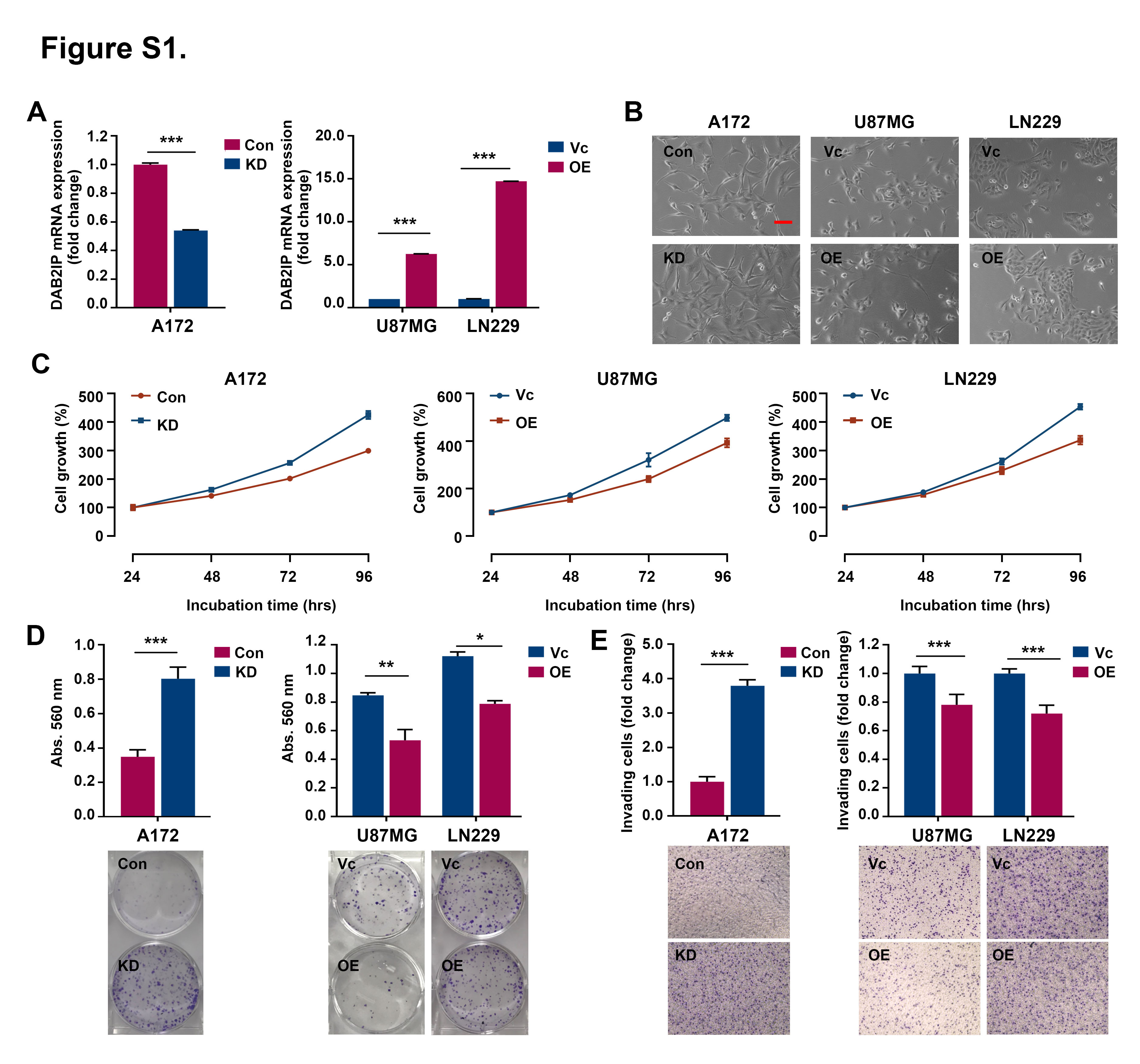

Supplement: Supplementary file 2 — Supplemental Figure 1. [file 41419_2023_5967_MOESM2_ESM.jpg]

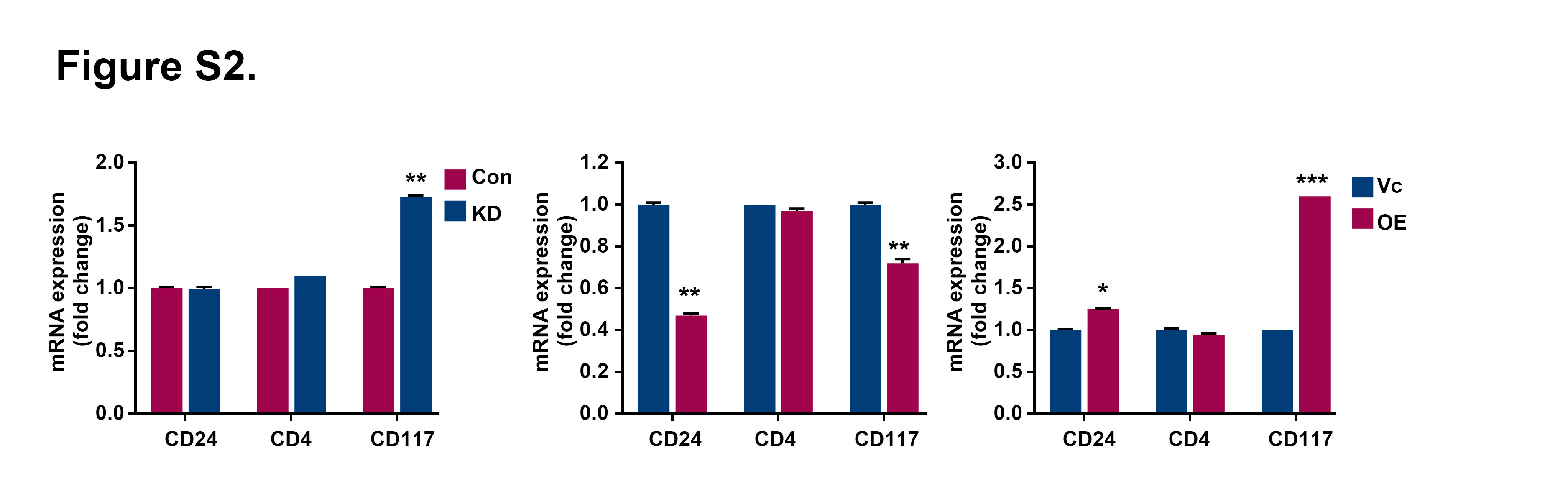

Supplement: Supplementary file 3 — Supplemental Figure 2. [file 41419_2023_5967_MOESM3_ESM.jpg]

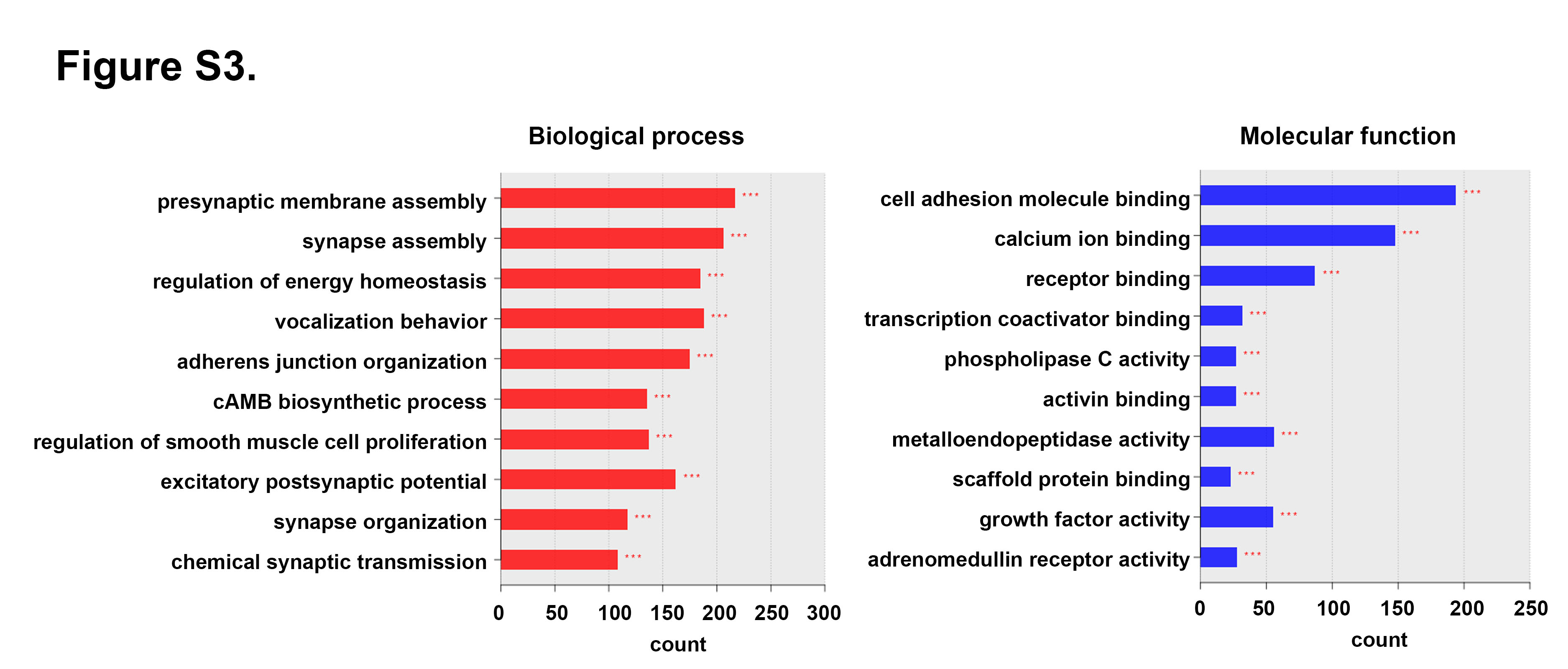

Supplement: Supplementary file 4 — Supplemental Figure 3. [file 41419_2023_5967_MOESM4_ESM.jpg]

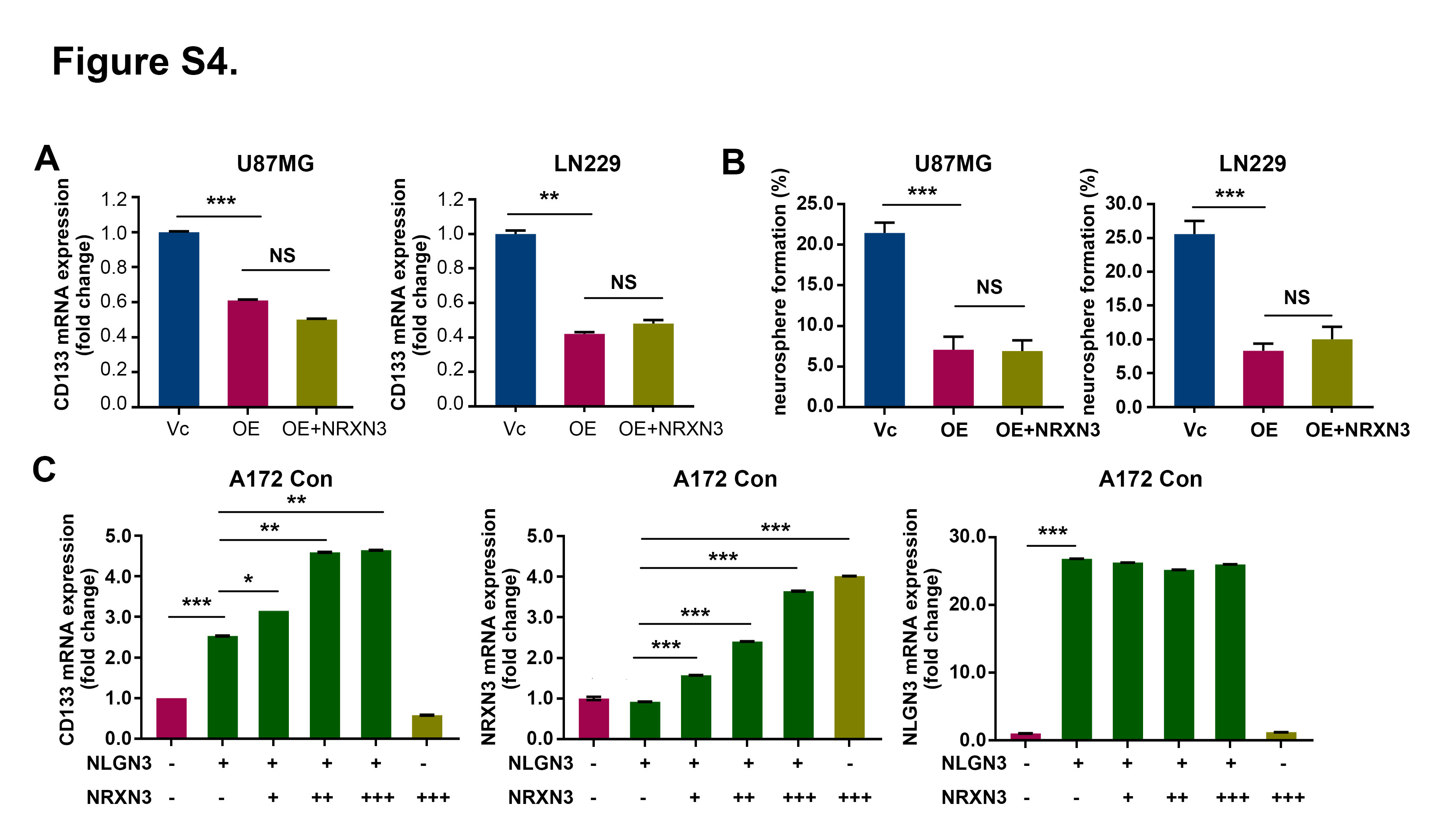

Supplement: Supplementary file 5 — Supplemental Figure 4. [file 41419_2023_5967_MOESM5_ESM.jpg]

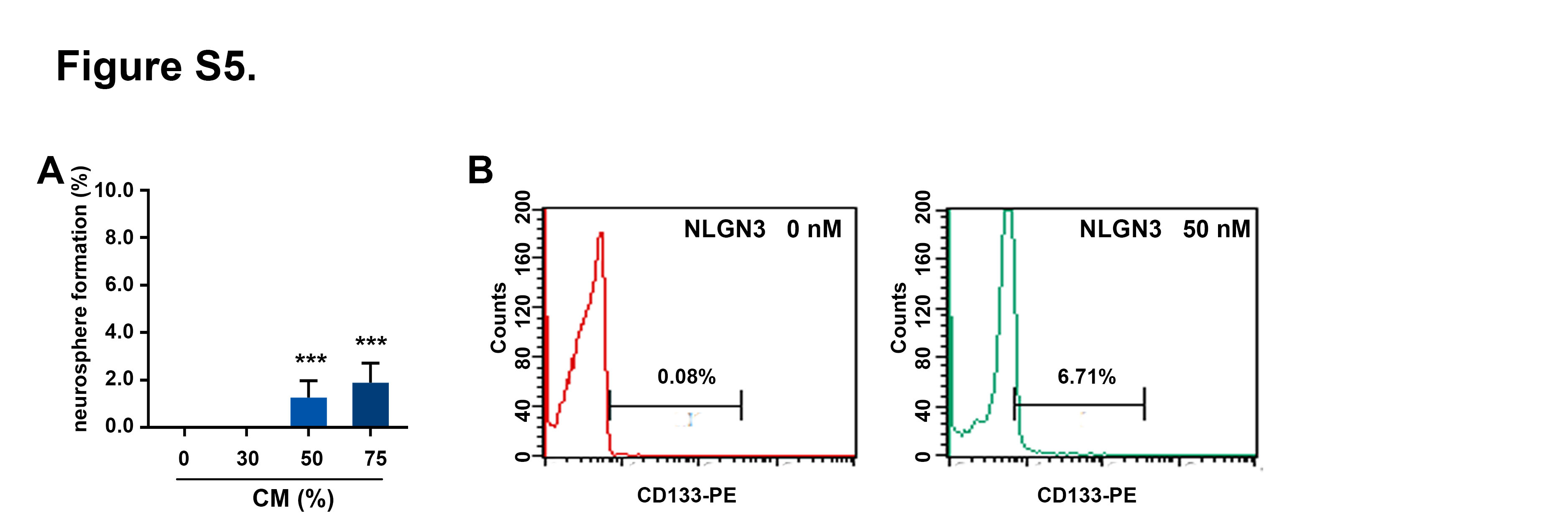

Supplement: Supplementary file 6 — Supplemental Figure 5. [file 41419_2023_5967_MOESM6_ESM.jpg]

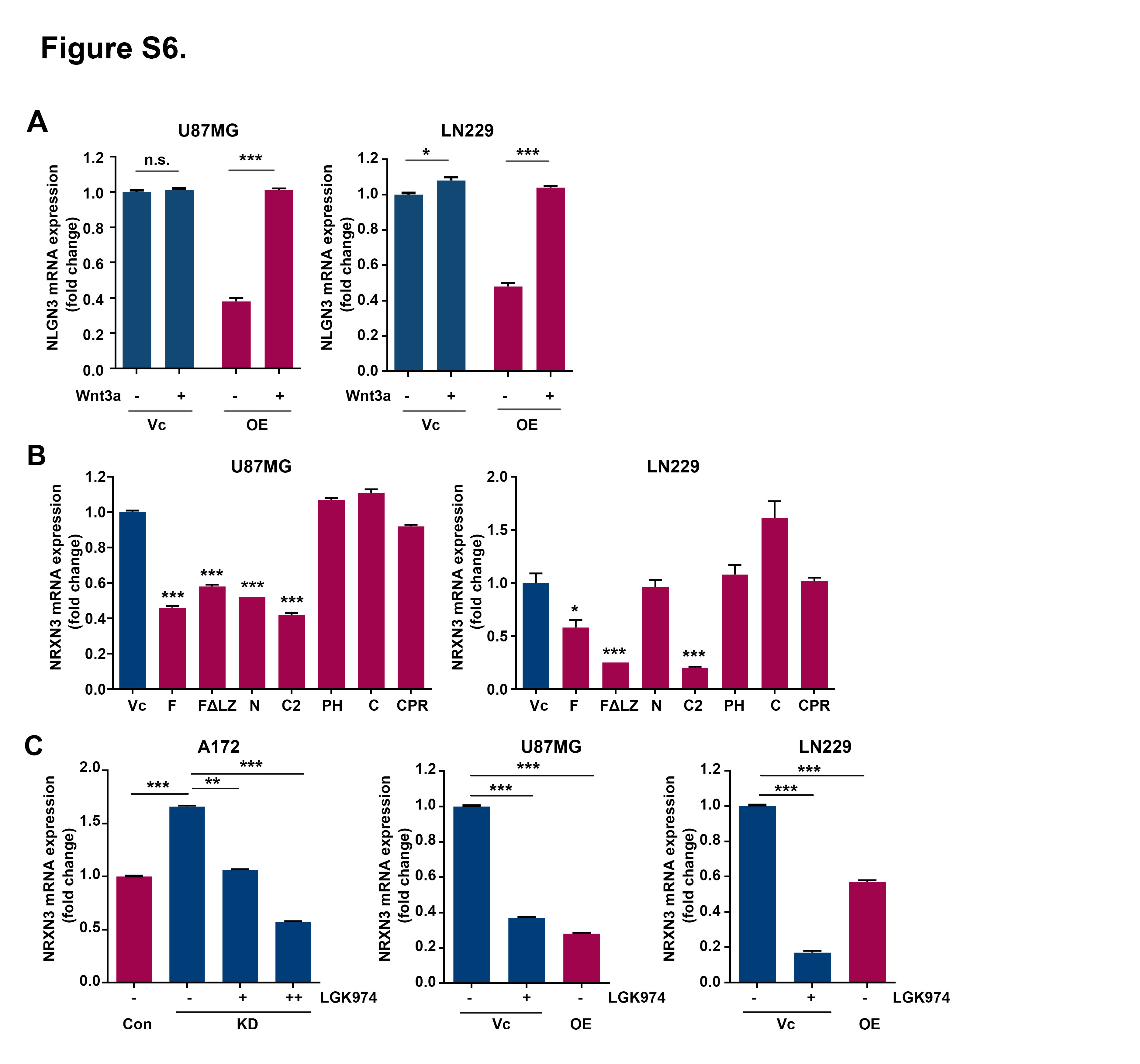

Supplement: Supplementary file 7 — Supplemental Figure 6. [file 41419_2023_5967_MOESM7_ESM.jpg]

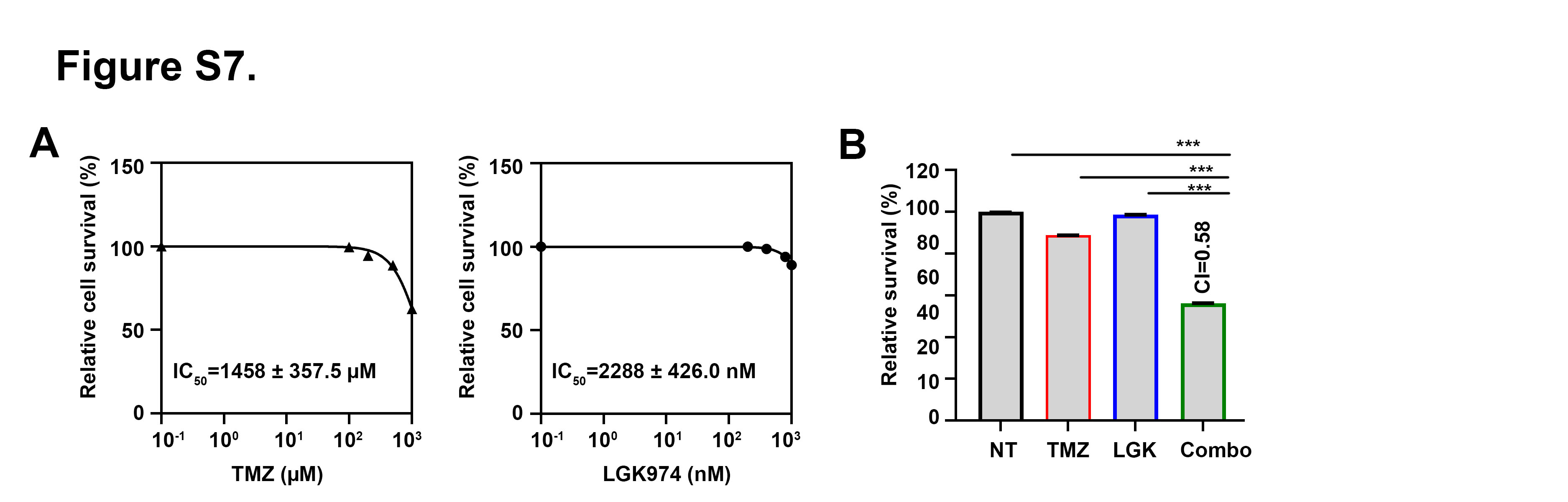

Supplement: Supplementary file 8 — Supplemental Figure 7. [file 41419_2023_5967_MOESM8_ESM.jpg]

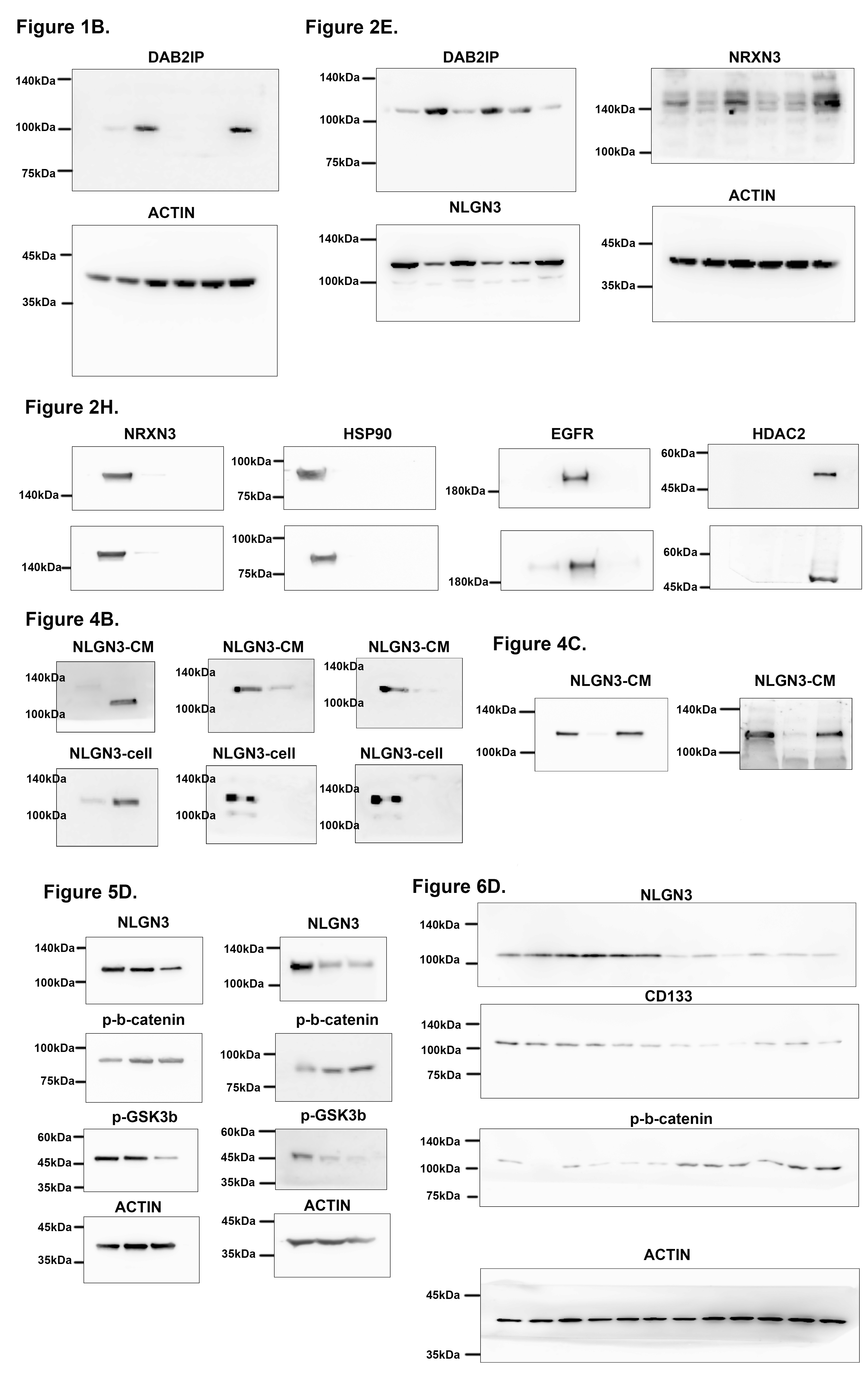

Supplement: Supplementary file 10 — Original Western blot [file 41419_2023_5967_MOESM10_ESM.tif]
