# Supplementary material for: Targeting Wnt/β-catenin-mediated upregulation of oncogenic NLGN3 suppresses cancer stem cells in glioblastoma
Source: Cell Death Dis. 2023 Jul 13;14(7):423. doi: 10.1038/s41419-023-05967-x (PMC10344874; doi:10.1038/s41419-023-05967-x)
Supplement: Supplementary file 9 — Supplemental Figure Legends [file 41419_2023_5967_MOESM9_ESM.docx]

**Supplemental figure legends**

**Figure S1. Expression of DAB2IP is associated with malignant phenotype of GBM cell lines. A.** DAB2IP mRNA expression was validated after modulation of DAB2IP. A172 cell was transfected with pGIPZ-shControl (Con) or pGIPZ-shDAB2IP (KD) to knock-down DAB2IP whereas U87MG and LN229 cell was transfected with pcDNA3.1-vector (Vc) or pcDNA3.1-DAB2IP (OE) to overexpress DAB2IP. Means ± SD; Student’s two tailed t-test, ***p<0.001. **B.** Cell morphologies were compared after modulation of DAB2IP. Scale bar = 100 µm. **C.** Cells were seeded in a 96-well with concentration of 1,000 cells/well and cell growth was determined at the indicated time points by MTT assay as described in Materials and Methods. **D.** Cells were seeded in 6-well plates at a density of 1,000 cells per well and cultured for 10 days to compare colony formation in DAB2IP modulated cells. Cells were fixed and stained with 4% formaldehyde, 0.05% crystal violet in PBS. Absorbance at 590 nm was measured after dissolving with 10% acetic acid. Red and blue bars indicate DAB2IP-high and DAB2IP-low expression cell lines, respectively. Means ± SD; Student’s two tailed t-test, *p<0.05, **p<0.01, ***p<0.001. **E.** Cells were seeded onto Matrigel-coated Transwell chambers for 48 h and quantitative measurements of invade cells were determined. Means ± SD; n=5; Student’s two tailed t-test, ***p<0.001.

**Figure S2. DAB2IP affects the expressions of various cancer stem cell surface markers depends on cell types.** The expression levels of CD24, CD44, and CD117 mRNA were analyzed. Means ± SD; Student’s two tailed t-test, *p<0.05, **p<0.01, ***p<0.001.

**Figure S3. Gene ontology enrichment analysis of DEG by DAB2IP.** Top 10 terms of GO functional analysis were shown. *p<0.05, **p<0.01, ***p<0.001.

**Figure S4. NRXN3 acts as a helper of NLGN3 regulation.**

**A.** After restoration of NRXN3 expressions in U87MG OE (DAB2IP-high, NRXN3-low) and LN229 OE (DAB2IP-high, NRXN3-low) cells, CD133 mRNA expression was compared. Red and blue bars indicate DAB2IP-high and DAB2IP-low expression cell lines, green bar indicates NRXN3 restoration in DAB2IP-high cells, respectively. Means ± SD; One-way ANOVA, **p<0.01, ***p<0.001. **B.** After restoration of NRXN3 expressions in U87MG OE and LN229 OE cells, sphere forming abilities were compared. Means ± SD; One-way ANOVA, ***p<0.001. **C.** A172 Con cells were co-transfected with NLGN3 and increment amount of NRXN3, and CD133 mRNA expression was compared. Means ± SD; One-way ANOVA, *p<0.05, **p<0.01, ***p<0.001.

**Figure S5. Secreted NLGN3 plays a role in maintaining cancer stem cell properties of GBM cells. A.** A172 Con cells were cultured in ultra-low attachment plate under sphere forming culture condition for 14 days, and CM derived from A172 KD cells were added into sphere every 3 days. Means ± SD; One-way ANOVA, ***p<0.001. **B.** Cells treated with recombinant NLGN3 for 48 h were stained with PE-conjugated CD133 and analyzed by flow cytometry. PE-IgG was used as the negative control for gating and the labels indicated the percentage of each cell population.

**Figure S6. DAB2IP regulates NRXN3 expression through Wnt/β-catenin signaling pathway. A.** Cells were transfected with various functional domains of DAB2IP and NRXN3 mRNA expressions were determined. Means ± SD; One-way ANOVA, *p<0.05, ***p<0.001. **B.** DAB2IP-low and DAB2IP-high cells were treated with Wnt3a (100 ng/mL) for 12 hours and NLGN3 mRNA expression was compared. Means ± SD; One-way ANOVA, *p<0.05, ***p<0.001. **C.** DAB2IP-low cells (A172 KD, U87MG Vc, and LN229 Vc) were treated with LGK974 and NRXN3 mRNA expression was compared. Means ± SD; One-way ANOVA, *p<0.05, **p<0.01, ***p<0.001.

**Figure S7. DAB2IP has no effect on the expressions of other NLGN family members.** The expression levels of NLGN3 family containing NLGN1, NLGN2, NLGN4X, and NLGN5 mRNA in DAB2IP modulated cells were analyzed. Red and blue bars indicate DAB2IP-high and DAB2IP-low expression cell lines, respectively. Means ± SD; n=3.

**Figure S8. GL261 has resistance to TMZ treatment. A.** GL261 cells were treated with increment doses of TMZ or LGK974as indicated for 48h, and IC_50_ was determined with cytotoxicity results assessed by MTT assay. Percent survival curves after treatment was constructed by plotting the cell survival whose values indicate mean ± SD (n=12). **B.** GL261 cells were seeded in a 96-well and treated with TMZ (500 µmol/L), LGK974 (400 nmol/L), or combination for 48h and cell viability was determined by MTT assay. Drug synergistic effect was determined based on CI. CI<1, synergistic; CI=1, additive; CI>1, antagonistic effect. NT, nontreatment; TMZ, temozolomide; LGK, LGK974; Combo, TMZ and LGK974 combination treatment. Means ± SD; One-way ANOVA, ***p<0.001.
